# Prediction of cardiovascular risk using machine-learning methods. Sex-specific differences

**DOI:** 10.3389/fcvm.2025.1579947

**Published:** 2025-06-19

**Authors:** Sara Castel-Feced, Isabel Aguilar-Palacio, Sara Malo, Juan González-García, Lina Maldonado, María José Rabanaque-Hernández

**Affiliations:** ^1^ Fundación Instituto de Investigación Sanitaria Aragón (IIS Aragón), Zaragoza, Spain; ^2^ Department of Employment, Science and Universities of the Government of Aragón (Spain), GRISSA Research Group, Zaragoza, Spain; ^3^ Department of Employment, Science and Universities of the Government of Aragón (Spain), Institute for Health Research Aragón (IIS Aragón), Zaragoza, Spain; ^4^ Network for Research on Chronicity, Primary Care, Health Promotion (RICAPPS), ISCIII, Madrid, Spain; ^5^ Department of Microbiology, Pediatrics, Radiology, and Public Health, University of Zaragoza, Zaragoza, Spain; ^6^ Biocomputing Unit, Data Science for Health Services and Policy Research Aragon Health Sciences Institute (IACS), Zaragoza, Spain; ^7^ Department of Applied Economy, Universidad de Zaragoza, Zaragoza, Spain

**Keywords:** machine learning, cardiovascular disease, adherence to treatment, random forest, XGBoost

## Abstract

**Background:**

Machine learning (ML) algorithms offer some advantages over traditional scoring systems to assess the influence of cardiovascular risk factors (CVRFs) on the risk of major cardiovascular event (MACE), and could be useful in clinical practice. These algorithms can also be trained using a growing body of real world data (RWD). The aim of the study was to evaluate the MACE risk applying the XGBoost and Random Forest ML algorithms to RWD, stratifying the study population by sex, comparing the outcomes of these two algorithms.

**Methods:**

The follow-up period of the study was from 2018 to 2020. For each algorithm, 3 models were generated, including age and different combinations of three groups of variables: blood test and blood pressure measurements; CVRFs; and medication adherence.

**Results:**

In this study, 52,393 subjects were included, of whom 581 suffered a MACE. The incidence of MACE was 1% in women and 1.3% in men. The most prevalent CVRF was hypertension, followed by hypercholesterolaemia in both sexes. Adherence to treatment was highest for antihypertensives and lowest for antidiabetics. In all models age was the greatest relative contributor to the risk of MACE, followed by adherence to antidiabetics. Adherence to treatment proved to be an important variable in the risk of having a MACE. Moreover, similar performance was found for RF and XGBoost algorithms.

**Conclusion:**

These findings support the use of ML to assess cardiovascular risk and guide personalized prevention strategies in primary care settings.

## 1 Introduction

Cardiovascular disease (CVD) is one of the leading causes of death and disability. It is estimated that CVD accounts for approximately 17.9 million deaths per year worldwide, and one-third of these deaths occur prematurely in people under the age of 70 (1). Some cardiovascular risk factors (CVRF) can be controlled, and CVD prevention guidelines highlight the importance of early diagnosis and intervention in high-risk individuals to prevent CVD mortality and morbidity (2). Lifestyle changes are among the most important primary prevention interventions. If these prove insufficient, pharmacological preventive treatment, selected according to the individual's overall cardiovascular risk, is indicated.

Different risk estimation tools are widely applied and recommended by CVD prevention guidelines to identify at-risk individuals who should be targeted for both behavioral and pharmacological primary prevention. These tools, which include the Framingham Risk Score and the Systematic Coronary Risk Evaluation (SCORE), estimate the individual's overall cardiovascular risk based on the individual contributions of multiple CVRFs, but are not without limitations (3–6). First, these tools have been developed for specific populations and, therefore, have limited generalizability for predicting risk in other populations and countries. Second, methodological limitations of these approaches include (i) the fact that they are based on simple regression fitting approaches and cannot assume a nonlinear relationship between predictors and outcomes; (ii) correlation between variables, and (iii) the risk of overfitting.

Furthermore, although pharmacological treatment and its adherence are related to cardiovascular risk (7–9), the aforementioned tools do not consider whether subjects are being treated for any CVRF or whether they correctly adhere to their treatment. While this issue can be addressed thanks to the growing availability of medical data generated in daily clinical practice (10), incorporation of these data in this context remains challenging, and requires an initial data-cleaning process. Finally, the incidence of CVRFs, and how they interact and are controlled, differs between men and women (2, 11–14) and therefore CVRFs should be analysed separately for each sex.

To improve the accuracy of traditional scores to overcome some of the aforementioned limitations, machine learning (ML) techniques have been applied and tested in several cohorts to identify individuals with high cardiovascular risk (3, 6, 15–17). ML techniques use routinely collected clinical data, as well as other data such as claims data, to train models to learn patterns that are later applied to the prediction of other variables. The techniques used include ensemble methods, which enable a kind of supervised ML, and include bagging and boosting methods that combine multiple decision trees to reach a decision (18). One of the most commonly used bagging methods is Random Forest (RF), whereby multiple decision trees are learned in parallel and the final prediction is based on the most frequent answer (15, 18). Boosting models, in contrast, train multiple individual models sequentially, with each model correcting the errors of its predecessor. Two well-known boosting methods are AdaBoost and XGBoost.

ML techniques have shown great promise in calculating CVD risk in different cohorts, improving upon the results obtained using traditional scoring methods. In this study, we compared the prediction of cardiovascular risk using ML methods applied to men and women together vs. separately, and analysed the influence of different traditional CVRFs together with medication adherence when included in these algorithms.

## 2 Materials and methods

### 2.1 CARhES cohort and data source

This longitudinal cohort study was conducted using the CArdiovascular Risk factors for hEalth Services research (CARhES) cohort (19). This dynamic open cohort has been followed since 2017, and includes all individuals aged 16 and above registered as users of the public health system in Aragón, a Spanish region with about 1.3 million inhabitants that are overwhelmingly attended to by the public health system. Participants had at least one of the following CVRFs: hypertension, hypercholesterolaemia, or diabetes mellitus (DM). Hypertension was identified based on a medical diagnosis of hypertension. DM and hypercholesterolaemia were identified based on a medical diagnosis of DM or hypercholesterolaemia and/ or a prescription of at least one antidiabetic, or lipid-lowering drug during the study period. The CARhES cohort consisted of 446,998 individuals (50.64% female), of whom 252,508 had hypertension (56.5%); 332,644 had hypercholesterolaemia (74.4%) and 96,709 had DM (21.6%).

All information necessary to identify patients who met the inclusion criteria was obtained from BIGAN (20), a health data hub that gathers data from the Aragon public health service and makes this information available for research purposes upon request. Data from this cohort were stored in several databases: the BDU (health system users database), which provides information on age and affiliation to the Aragón public health system; the minimum basic dataset database, which gathers data on hospital discharge; the primary care database, which records information from patients who attend a primary health care centre; GMA (morbidity adjusted groups), which records information on all medical diagnoses available in primary healthcare and in the minimum basic dataset database; the emergency database, which stores diagnostic and procedural information on patients processed via the hospital emergency system; the e-prescription database, which records all pharmacological treatments prescribed to patients; and the pharmacy claims database, which gathers information about medication dispensed in pharmacies to each patient. All data in these databases are pseudonymized using a unique code that links patient information across the different data sources but prevents personal identification.

The GMA database was queried to identify subjects with a medical diagnosis corresponding to any of the 3 CVRFs of interest. Pharmacological treatments that corresponded to the following ATC codes and were prescribed to patients were extracted from the e-prescription database: A10 (diabetes); C02, C03, C07, C08, and C09 (hypertension); and C10 (hypercholesterolaemia).

### 2.2 Study design

The present study was conducted within the CARhES cohort considering all individuals who were in the cohort in 2017. They were followed from 2018 until 2020 to identify if they suffered a major cardiovascular event (MACE).

### 2.3 Inclusion and exclusion criteria

**Figure 1:**
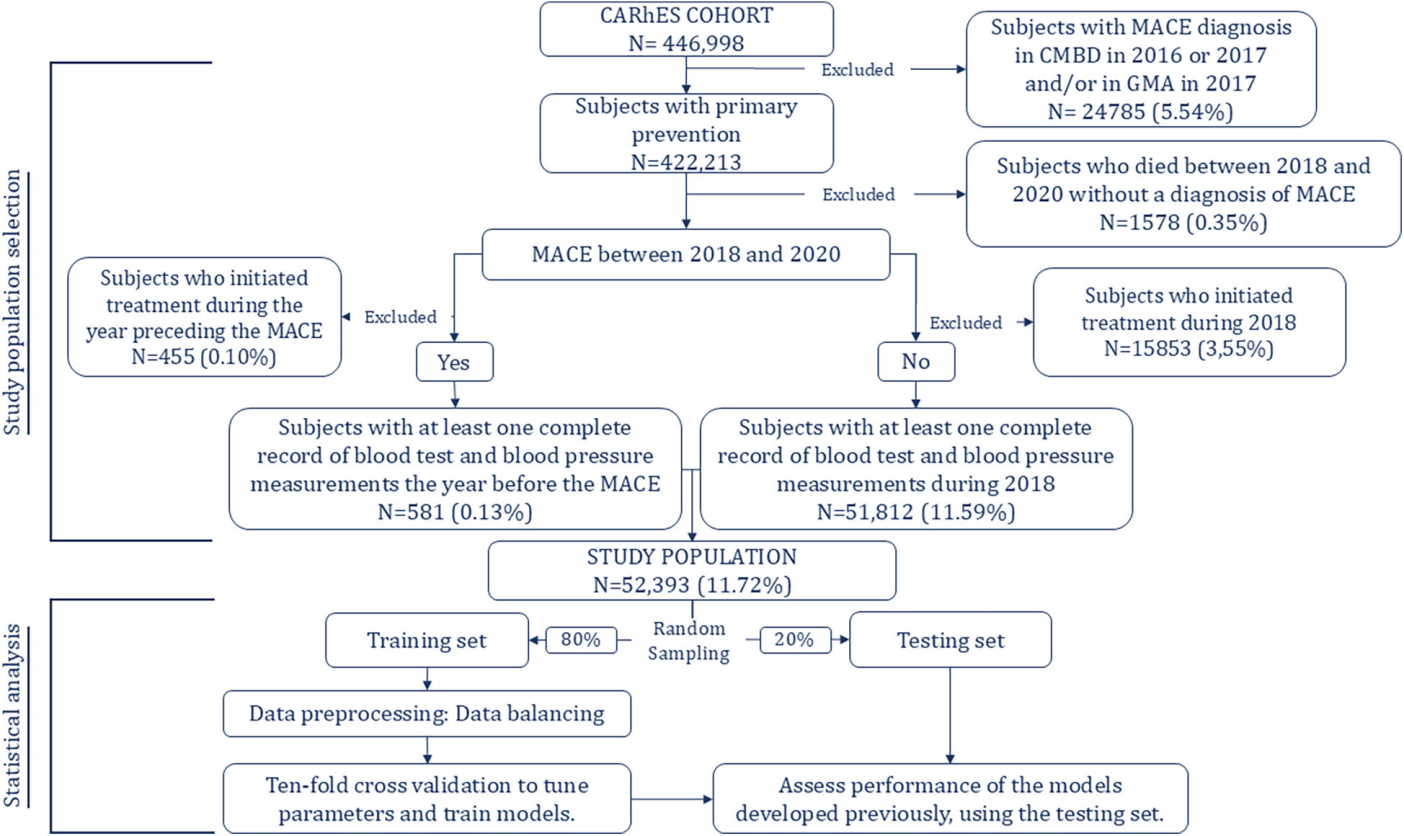
Study population selection and model development.

The process of selecting patients from the CARhES cohort to participate in the present study is depicted in Figure 1. First, as we focused on subjects with primary prevention, we excluded those with a diagnosis of MACE in the minimum basic dataset database and/or in the GMA before 2018.

Also, they were excluded patients who died during the follow-up period, for whom MACE was not recorded as the cause of the death. Next, we identified subjects in primary prevention who had experienced MACE between 2018 and 2020. Of those who had, subjects who began treatment corresponding to any of the three pharmacological groups of interest during the year preceding the event were excluded. Of those who did not experience MACE during the follow-up period, we excluded those who began treatment during 2018. It was done based on the criteria established for the follow up period, explained below. Finally, among those who experienced MACE we included those for whom blood test and blood pressure data were available for the year preceding the event and, among those who did not experience MACE, we included those for whom blood test and blood pressure data were available for the year 2018.

### 2.4 Study variables

Variables included in the present study were age, blood test- and blood pressure-related parameters, CVRFs, and adherence to medication taken for CVRFs. The variables included and the corresponding data sources are summarised in Supplementary Table S1.

Adherence to antihypertensive, antidiabetic, or lipid-lowering drugs was calculated separately for each subject using the Proportion of Days Covered (PDC). Adherence was calculated as a percentage using data from the year 2018 for those who did not experience an event, and using data from the year preceding the event in all other cases. PDC is an index calculated as the number of days covered by the medicines dispensed by the pharmacy divided by the number of days that the subject should have had covered. In this study, the denominator for PDC was 365 days. The number of days covered were calculated based on the Defined Daily Dose (DDD) dispensed to each subject. However, a previous study by our group (9) showed that the use of a surrogate value for the daily dose of each drug, calculated based on the median time between prescriptions of the same drug in users classified as persistent, provided more accurate results. Therefore, in the present study surrogate values for daily doses were used. For example, for statins we always used a DDD of 28 rather than the value of 37.3 used in other studies.

The primary outcome in this study was MACE incidence during follow up, from 2018 to 2020. Episodes were identified in the minimum basic dataset database and the emergency database. An episode was considered a MACE if the first diagnosis in the minimum basic dataset database corresponded to one of the following ICD-10 codes: I21, I60-I63, corresponding to myocardial infarction, nontraumatic subarachnoid haemorrhage, nontraumatic intracerebral haemorrhage, other nontraumatic intracranial haemorrhage, and acute ischemic stroke, respectively. In the emergency database, episodes considered MACE were those with the same diagnosis, corresponding to ICD-9 codes 410 and 430–433, and that caused death.

### 2.5 Analysis

Random Forest (RF) and XGBoost were used to determine the utility of different variables to predict the risk of MACE. Random Forest is an ensemble learning method that builds multiple decision trees and combines their predictions to improve accuracy and prevent overfitting. XGBoost (Extreme Gradient Boosting) is a powerful machine learning algorithm that uses boosting, a technique where models are trained sequentially to correct errors made by previous ones, leading to better performance and generalization. Both were applied stratified by sex to age plus 3 different groups of variables:

• Model 1: Age, blood test and blood pressure measurement, cardiovascular risk factors, and medication adherence.
• Model 2: Age, blood test and blood pressure measurement, and medication adherence.
• Model 3: Age, cardiovascular risk factors, and medication adherence.

As shown in Figure 1, and as usually done when using these techniques, the study population was randomly split into two groups: 80% of the sample was assigned to the training group and the remaining 20% to the testing group. To train and tune the models 10-fold cross validation was applied to the training dataset to avoid overfitting. For both algorithms, hyperparameters were determined using a grid search in the 10-fold cross validation of the training set to identify values that led to optimal performance.

When event incidence is low, data are considered to be imbalanced. We observed a MACE incidence of 1.12%, indicating that the data were highly imbalanced. To solve this problem, the Random Over Sampling Examples (ROSE) method with replacement was used to oversample the minority class and balance the data in the training set (21–24). To avoid poor estimates of model performance, the resampling process was applied to each of the 10 subsamples created during the cross-validation process irrespective of the other subsamples. Other resampling methods were applied, but the results obtained with ROSE were the best, therefore, only these results are presented in this article.

The performance of the models was assessed using the test set, and Youden's Index used to establish the optimal threshold for classification. In cases of imbalanced data, certain measures such as accuracy, positive predictive value, and negative predictive value can be markedly altered. Therefore, to assess the performance of the models created we calculated four distinct parameters: (i) AUC, which evaluates the performance of binary classifiers; (ii) F1 score, which reflects the ability of the model to capture sensitivity and precision (i.e., to be accurate in the cases that it does capture); (iii) sensitivity, which indicates the proportion of cases classified as at high risk of an event; (iv) and specificity, which reflects the proportion of non-cases classified as such. Finally, the contribution of each variable to the prediction was extracted and standardized using a scale of 0–1 for ease of comparability. This was achieved by calculating the feature importance score for each variable, which reflects its contribution to reducing the model's error. In Random Forest, this is typically done by measuring the decrease in Gini impurity or entropy, while in XGBoost, the contribution is calculated through the gain, which indicates the improvement in prediction accuracy attributed to each variable.

### 2.6 Ethical approval and consent to participate

The present study was approved by the Clinical Research Ethics Committee of Aragon (CEICA) in 2021 (project identification code PI21/148).

## 3 Results

### 3.1 Descriptive analysis of total population and by sex

Of the 52,393 individuals included in the present study, 57.3% were women, and the mean age was 70.2 years. Female participants were older than their male counterparts (mean age, 71.6 and 68.3 years, respectively) (Table 1).

**Table 1:** Descriptive statistics for study population.

Variables	Units	Total *N* = 52,393	MEN *N* = 22,383	WOMEN *N* = 30,010	*P* value
Age	mean (SD)	70.2 (12.8)	68.3 (12.6)	71.6 (12.8)	<0.001
Cardiovascular risk factors					
DM	*N* (%)	14,181 (27.1)	7162 (32.0)	7019 (23.4)	<0.001
Hypertension	*N* (%)	38,253 (73.0)	15,964 (71.3)	22,289 (74.3)	<0.001
Hypercholesterolaemia	*N* (%)	37,316 (71.2)	15,877 (70.9)	21,439 (71.4)	0.209
Number of CVRFs	*N* (%)				<0.001
1		22,508 (43.0)	9406 (42.0)	13,102 (43.7)	
2		22,413 (42.8)	9334 (41.7)	13,079 (43.6)	
3		7,472 (14.3)	3,643 (16.3)	3,829 (12.8)	
Blood test and blood pressure measurements					
Total cholesterol levels (mg/dl)	mean (SD)	195 (36.1)	186 (35.5)	201 (35.1)	0.000
HDL cholesterol levels (mg/dl)	mean (SD)	53.7 (13.4)	48.8 (11.6)	57.3 (13.5)	0.000
LDL cholesterol levels (mg/dl)	mean (SD)	118 (31.5)	114 (31.7)	121 (31.1)	<0.001
Blood glucose levels (mg/dl)	mean (SD)	104 (24.8)	107 (26.5)	101 (23.1)	<0.001
Systolic blood pressure (mm Hg)	mean (SD)	133 (15.8)	134 (15.4)	133 (16.2)	<0.001
Diastolic blood pressure (mm Hg)	mean (SD)	76.8 (13.9)	77.8 (16.5)	76.0 (11.4)	<0.001
Medication adherence, PDC					
Antihypertensives	mean (SD)	58.3 (44.0)	57.5 (44.7)	58.9 (43.6)	<0.001
Antidiabetics	mean (SD)	17.3 (33.4)	21.0 (36.1)	14.5 (31.0)	<0.001
Lipid-lowering drugs	mean (SD)	38.5 (42.0)	40.1 (42.5)	37.3 (41.5)	<0.001
MACE characteristics					
Incidence	*N* (%)	581 (1.1)	282 (1.3)	299 (1.0)	0.005
Diagnosis	*N* (%)				0.011
Acute Ischemic stroke		334 (57.5)	150 (53.2)	184 (61.5)	
Myocardial infarction		152 (26.2)	91 (32.3)	61 (20.4)	
Nontraumatic subarachnoid haemorrhage		14 (2.4)	4 (1.4)	10 (3.3)	
Nontraumatic intracerebral haemorrhage		57 (9.8)	24 (8.5)	33 (11.0)	
Other nontraumatic intracranial haemorrhage		24 (4.1)	13 (4.6)	11 (3.7)	

SD, standard deviation; N, number; DM, diabetes mellitus; HDL, high density lipoprotein; LDL, low density lipoprotein; MACE, major cardiovascular event; PDC, proportion of days covered.

For both sexes, the most prevalent CVRF was hypertension, followed by hypercholesterolaemia. The proportions of individuals with 1 and 2 CVRFs were similar in both sexes. Around 40% of participants had just one CVRF.

Mean values of total, HDL, and LDL cholesterol were higher in women than men, and mean blood glucose, systolic blood pressure (SBP), and diastolic blood pressure (DBP) were slightly higher in men than women.

Adherence to treatment was highest for antihypertensives and lowest for antidiabetics, both in the total population and after stratifying by sex. For antidiabetics and lipid-lowering drugs, men showed higher mean adherence, but also greater dispersion. For antihypertensives, mean adherence was higher in women but dispersion higher in men.

A MACE was experienced by 581 (1.1%) participants: 282 men and 299 women (Table 1), in 3 years of follow up. In 12 cases (8 men, 4 women) the event resulted in death. The most common MACE was stroke, representing 57.5% of all events, followed by myocardial infarction (MI) (26.2%). Stratifying by sex, stroke was more frequent in women than men (61.5% and 53.2%, respectively), while MI was more frequent in men than women (32.3% and 20.4%, respectively).

### 3.2 Characteristics of individuals with MACE

Of the total number of MACEs, 51% were experienced by women, although the incidence of MACE was higher in men. Mean age was higher among individuals who experienced a MACE: 78.9 and 70.1 years in individuals who did and did not experience MACE, respectively (Table 2).

**Table 2:** Descriptive statistics for patients with MACE.

Variables	Units	No MACE *N* = 51,812	MACE *N* = 581	*P* value	OR [95% CI]
Age	mean (SD)	70.1 (12.8)	78.9 (9.92)	<0.001	1.07 [1.07;1.08]
Sex	*N* (%)			0.005	
Men		22,101 (42.7)	282 (48.5)		Ref.
Women		29,711 (57.3)	299 (51.5)		0.79 [0.67;0.93]
Cardiovascular risk factors					
Diabetes	*N* (%)	13,952 (26.9)	229 (39.4)	<0.001	1.77 [1.49;2.09]
Hypertension	*N* (%)	37,767 (72.9)	486 (83.6)	<0.001	1.90 [1.53;2.38]
Hypercholesterolaemia	*N* (%)	36,906 (71.2)	410 (70.6)	0.761	0.97 [0.81;1.16]
Number of CVRF	*N* (%)			<0.001	
1		22,330 (43.1)	178 (30.6)		Ref.
2		22,151 (42.8)	262 (45.1)		1.48 [1.23;1.80]
3		7,331 (14.1)	141 (24.3)		2.41 [1.93;3.01]
Blood test and blood pressure tests					
Total cholesterol levels (mg/dl)	mean (SD)	195 (36.1)	187 (35.2)	<0.001	0.99 [0.99;1.00]
HDL cholesterol levels (mg/dl)	mean (SD)	53.7 (13.4)	50.9 (12.9)	<0.001	0.98 [0.98;0.99]
LDL cholesterol levels (mg/dl)	mean (SD)	118 (31.5)	111 (30.6)	<0.001	0.99 [0.99;1.00]
Blood glucose levels (mg/dl)	mean (SD)	104 (24.6)	109 (38.0)	<0.001	1.01 [1.00;1.01]
Systolic blood pressure (mm Hg)	mean (SD)	133 (15.8)	137 (16.6)	<0.001	1.02 [1.01;1.02]
Diastolic blood pressure (mg Hg)	mean (SD)	76.8 (13.9)	75.0 (10.7)	<0.001	0.98 [0.98;0.99]
Medication adherence, PDC					
Antihypertensives	mean (SD)	58.2 (44.1)	66.2 (40.9)	<0.001	1.00 [1.00;1.01]
Antidiabetics	mean (SD)	17.2 (33.4)	27.0 (39.2)	<0.001	1.01 [1.01;1.01]
Lipid-lowering drugs	mean (SD)	38.5 (42.0)	38.7 (42.0)	0.908	1.00 [1.00;1.00]

MACE, major cardiovascular event; SD, standard deviation; N, number; DM, diabetes mellitus; HDL, high density lipoprotein; LDL, low density lipoprotein; PDC, proportion of days covered.

The frequencies of DM and hypertension were higher among individuals who experienced a MACE. There were no significant differences in the proportion of patients with hypercholesterolaemia between individuals with or without MACE. Moreover, those who experienced MACE more frequently presented 2 or 3 CVRFs, and those who did not more frequently presented 1 CVRF.

We observed no difference in adherence to lipid-lowering drugs between individuals with or without a MACE. Those who did experience a MACE were more adherent to antihypertensive and antidiabetic drugs.

### 3.3 Cardiovascular risk prediction

#### 3.3.1 Models built with random forest

After stratifying by sex using the RF method, model performance (measured by AUC) was higher in women than in men (Table 3). For women, the highest performance was obtained for model 3, when using CVRF variables together with adherence. In contrast, for men, performance was slightly higher for models 1 and 2 than for model 3. Differences in performance between models were higher in women than in men.

**Table 3:** Performance metrics for random forest and XGBoost models.

Model	AUC	Youden's index	F1 score	Sensitivity	Specificity
Random forest					
Men					
MODEL 1	0.70	0.50	0.77	0.62	0.69
MODEL 2	0.70	0.52	0.76	0.61	0.71
MODEL 3	0.69	0.54	0.77	0.62	0.71
Women					
MODEL 1	0.77	0.64	0.71	0.66	0.75
MODEL 2	0.76	0.62	0.81	0.69	0.75
MODEL 3	0.79	0.53	0.84	0.72	0.75
XGBOOST					
Men					
MODEL 1	0.70	0.53	0.78	0.64	0.71
MODEL 2	0.70	0.51	0.79	0.65	0.68
MODEL 3	0.69	0.52	0.79	0.65	0.66
Women					
MODEL 1	0.74	0.58	0.89	0.80	0.56
MODEL 2	0.76	0.54	0.80	0.67	0.81
MODEL 3	0.79	0.50	0.81	0.69	0.78

Model 1 includes the variables age, CVRFs, adherence, and blood test and blood pressure measurements. Model 2 includes age, adherence, and blood test and blood pressure measurements. Model 3 includes age, CVRFs, and treatment adherence.

AUC, area under the curve; CVRF, cardiovascular risk factor.

Of the models built for men, model 3 provided the highest F1 score, sensitivity, and specificity, although it had the lowest AUC. For women, the highest F1 score and sensitivity were achieved with model 3, while all models reached a specificity of 0.75. As also observed with AUC, differences between F1 score, sensitivity, and specificity were smaller in the male population compared to the female population.

##### 3.3.1.1 Relative contributions of variables

**Figure 2:**
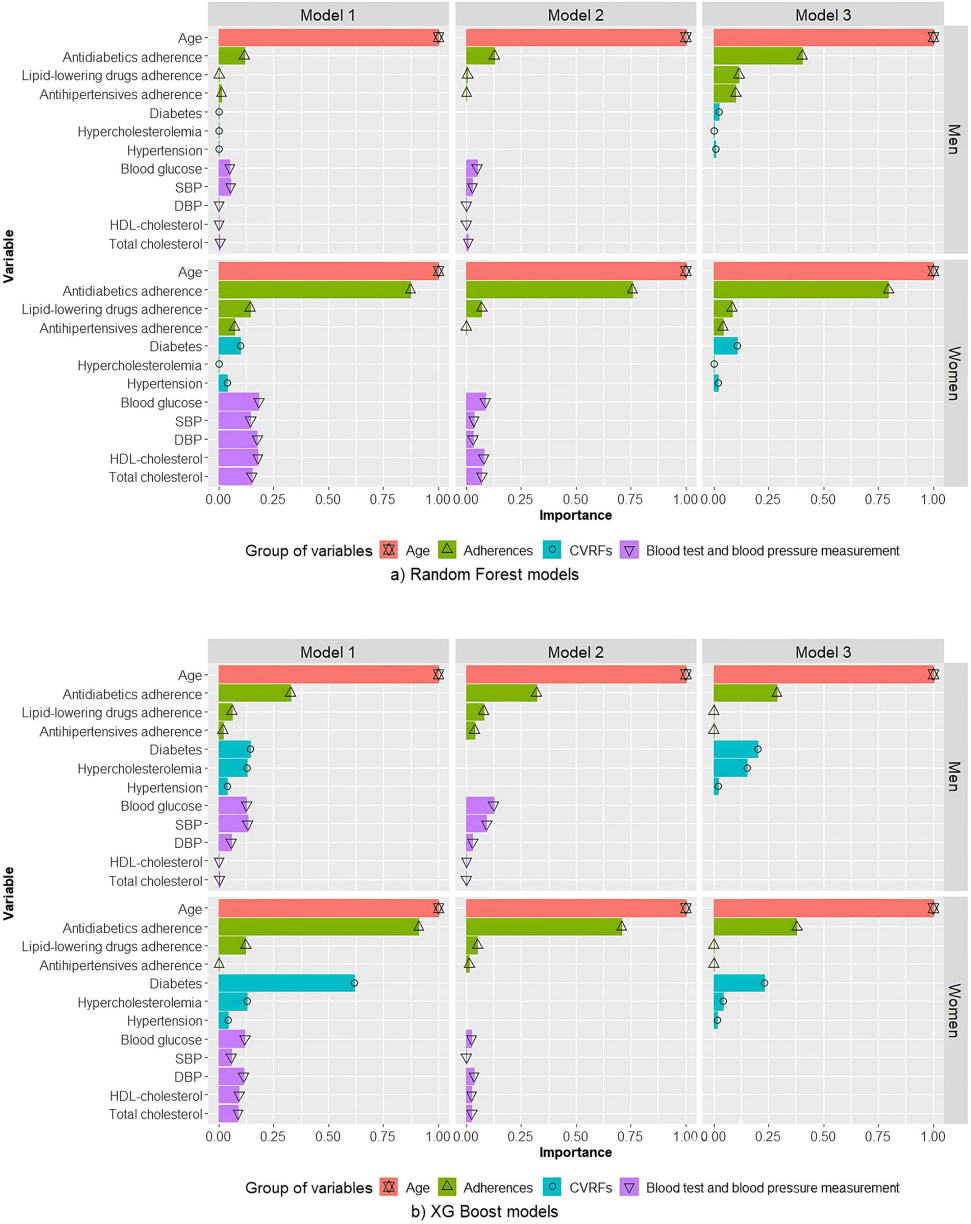
Relative contributions of variables in random forest **(a)** and XGBoost **(b)** models for men and women. Model 1 includes the variables age, CVRFs, adherence, and blood test and blood pressure measurements. Model 2 includes age, adherence, and blood test and blood pressure measurements. Model 3 includes age, CVRFs, and treatment adherence. SBP, systolic blood pressure; DBP, diastolic blood pressure; HDL-cholesterol, high density lipoprotein cholesterol; CVRF, cardiovascular risk factor.

In all RF models, for both men and women, age was the variable that contributed most to the risk of MACE (Figure 2a).

For men, age was the variable for which the greatest contribution to risk of MACE was observed, followed by antidiabetic adherence. The contribution of antidiabetic treatment adherence in models 1 and 2 was much smaller than the contribution of age. Furthermore, the contribution of antidiabetic treatment adherence in model 3 was higher than in models 1 and 2, but not as high as observed for women.

For women, in terms of relative contributions to the risk of MACE, age was closely followed by antidiabetic treatment adherence. All other variables contributed less. For model 1, blood test and pressure measurements variables were greater contributors than a diagnosis of hypertension, DM, or hypercholesterolaemia, and than adherence to lipid-lowering drugs or hypertension.

#### 3.3.2 Models built with XGBoost

Models developed for the male population using XGBoost achieved AUC levels comparable to those of the RF models. F1 score and sensitivity were higher than those obtained with RF models, while specificity was lower for models 2 and 3, but higher for model 1.

In models developed for the female population using XGBoost (Table 3), AUC was comparable to that of the RF models for models 2 and 3, while it was lower for model 1 compared to the corresponding RF model. F1 score and sensitivity were highest for model 1, while specificity was highest for model 2. Compared with the corresponding RF model, model 1 in XGBoost showed higher F1 score and sensitivity, but not specificity.

##### 3.3.2.1 Relative contributions of variables

In models built using XGBoost, for both men and women, the variables that contributed most to a predicted high risk of MACE were age followed by antidiabetic treatment adherence (Figure 2b). For men, in XGBoost model 1, similar contributions were observed for DM and hypercholesterolaemia and for blood glucose and SBP. For women, contrary to that which was observed for RF models, in XGBoost model 1 DM was a much more important contributor than blood tests and blood pressure measurements, with an effect similar to that of antidiabetic adherence.

## 4 Discussion

In the present study we performed a descriptive analysis of CVRFs and MACE incidence using real-world data (RWD) from users of the Aragonese public health system. Using different combinations of predictive variables, we evaluated the abilities of two ML algorithms to predict MACE risk, and analysed the relative contributions of distinct CVR-related variables, stratifying the study population by sex.

The study population consisted of more women than men. The most prevalent CVRF was hypertension, and incidence of MACE was higher in men. The most frequent MACE in both sexes was stroke. The prevalence of DM and hypertension were higher among those who had experienced a MACE than those who had not. It was also found that medication adherence was higher in those with MACE than in those without. This could be explained by the fact that individuals with a worse health status may be more aware of their risk and show higher medication adherence (25).

Because the incidence, interactions, and control of CVRFs differ in men vs. women (11–14), we generated and evaluated three models for each sex using RF and XGBoost algorithms. In all models, for both sexes, age was the parameter that contributed most to a predicted high risk of MACE, followed closely by adherence to antidiabetics. Adherence to antidiabetics was a greater contributor to MACE in women than in men.

For both men and women, the contributions of individual variables to a predicted risk of MACE differed across models. In most models, age and adherence to antidiabetics were the main contributors, with the exception of XGBoost models for men, in which antidiabetic adherence was closely followed by DM in models 1 and 2, and by blood glucose in model 3.

Previous studies that include age as a predictive variable have consistently shown that this parameter has the greatest predictive power, suggesting that age is a key CVRF (3, 6, 17, 26). To the best of our knowledge, no studies based on ML methods published to date have considered cardiovascular treatment as a predictive variable, while those that consider adherence either measured this variable using questionnaires or did not consider adherence to antidiabetic treatments (3, 6, 22).

Our study identified adherence to antidiabetics as a key determinant of MACE in both sexes. Conversely, adherence to antihypertensives and lipid-lowering drugs showed little predictive power. Multiple studies have reported associations between adherence to antihypertensives and lipid-lowering drugs and the incidence of cardiovascular events (CVE) and all-cause mortality risk (27–30). In terms of influence on the risk of different types of CVE, adherence to antidiabetics is less well studied than adherence to lipid-lowering drugs and antihypertensives (23). In their systematic review, Mengying et al. (27) considered antidiabetics, antihypertensives and lipid-lowering drugs adherence, and found that all three were associated with a higher risk of CVE.

The aforementioned findings underscore the importance of proper pharmacological control of modifiable CVRFs to reduce the risk of CVE. Clinical guidelines propose controlling CVRFs in order to decrease this risk (2, 31). Previous research (32–34) has shown that adherence to these treatments is suboptimal, and the methods most commonly used to determine the risk of CVE do not include medication adherence as a predictive variable. There is also evidence (30) suggesting that a considerable number of CVEs are due to poor adherence to cardiovascular preventive treatments. Therefore, measuring adherence could maximize the efficacy of cardiac therapies in clinical settings.

Of the previously published studies similar to ours, analyses were performed without stratifying according to sex, and in most (3, 17, 26) sex was not identified as an important variable, with the exception of one study (6) in which sex was the second most important contributor to overall CVE risk. Although each study considered different variables, including lab results, blood pressure measurements, and socio-demographic factors, the importance of each varied across models and studies, as in the present study. Only in one study was blood glucose identified as the most important variable (17). Two studies identified SBP as the second most important variable (3, 26).

Studies have shown that models built using ML techniques can overcome certain limitations of traditional methods used to predict cardiovascular risk, as well as offering greater predictive power (3, 6, 17, 35). The models described in this study could be applied in clinical practice to assess the individual risk of MACE based on patient characteristics and medication adherence, thereby playing an important role in screening processes. This is particularly important given that primary-care-based interventions targeting individuals considered to be at high risk, based on their age or risk factors, appear to be effective in reducing the risk of CVE (36). Furthermore, these models can help orient the intervention and identify the most appropriate measures to take.

In addition to the advantages described above, ML techniques offer a variety of approaches to process large amounts of data to predict CVE incidence, thus allowing researchers and clinicians to select the algorithm that best suits their data or objectives.

### 4.1 Limitations and strengths

Some limitations of the present study should be noted. First, the incidence of MACE during the follow-up period was low, resulting in class-imbalanced data. This issue was addressed by applying the ROSE method to subsample the majority class. Second, the follow-up period was short, owing to the availability of data for the period 2018 to 2020 only. However, we feel that the size of the study population was sufficient to answer the research question. Finally, because this study was conducted using data extracted from administrative databases, some data were unavailable or were of insufficient quality to be included. Examples include smoking and physical activity data, which were recorded in very few subjects, and after quality control were deemed not to be reliable.

A key strength of the study is the fact that it was conducted with RWD, obtained from multiple data registries, enabling evaluation of the variables of interest in a real-world context. Our study is remarkable in that it includes data extracted from different levels of care from all individuals residing in Aragon, aged 16 and older, with any CVRF. Moreover, we used two different ML techniques, which integrate all available data and offer several advantages over earlier algorithms, as explained above, and compared the results obtained with each to determine the most accurate method. To our knowledge, few ML studies have examined the predictive power of treatment adherence, and those that have typically assess adherence by asking patients whether they are taking any medication, without considering whether this medication is prescribed by a doctor or whether the patient actually collects their medication from a pharmacy. Finally, our analysis considered two algorithms and different combinations of predictive variables, allowing us to identify the model that performed best in this particular study population and to evaluate the influence of different variables on MACE occurrence.

## 5 Conclusions

In the present study we found that, in all models and in both men and women, age was the variable that most contributed to the risk of CVE. Although this effect was greater in men than women. The next greatest contributor was adherence to antidiabetics, the effect of which was greater in women than in men. Comparison of distinct ML methods revealed comparable performance for RF and XGBoost algorithms. Our findings suggest that ML techniques offer a valuable means of analysing large amounts of data to help accurately assess the risk of MACE, and could ultimately be applied in CVD prevention programs in a personalised medicine context.

## Data Availability

The data analyzed in this study is subject to the following licenses/restrictions: All data used in this study pertain to the CARhES cohort. While these data are not publicly available due to their sensitive nature, interested researchers can nonetheless contact the CARhES cohort to request access. Requests to access these datasets should be directed to smalo@unizar.es or iaguilar@unizar.es.
