# Supplementary material for: Prediction of cardiovascular risk using machine-learning methods. Sex-specific differences
Source: Front Cardiovasc Med. 2025 Jun 19;12:1579947. doi: 10.3389/fcvm.2025.1579947 (PMC12222323; doi:10.3389/fcvm.2025.1579947)
Supplement: Supplementary file 1 [file Table1.docx]

Supplementary Material

Supplementary Table 1: Variables included in the study

| **GROUP** | **VARIABLE** | **DATABASE** | **VALUES/ UNITS** |
| --- | --- | --- | --- |
| **Age** | Age | BDU | Years |
| **CVRFs** | Hypertension | GMA | Yes/no |
|  | Hypercholesterolaemia | GMA +  e-prescription | Yes/no |
|  | Diabetes mellitus | GMA+  e-prescription | Yes/no |
| **Blood test and blood pressure measurement** | Total cholesterol | Primary care database | mg/dL |
|  | HDL-cholesterol | Primary care database | mg/dL |
|  | LDL-cholesterol | Primary care database | mg/dL |
|  | Blood glucose | Primary care database | mg/dL |
|  | Systolic blood pressure | Primary care database | mm Hg |
|  | Diastolic blood pressure | Primary care database | mm Hg |
| **Medication adherence** | Adherence to antihypertensives | Pharmacy claims | % |
|  | Adherence to lipid-lowering drugs | Pharmacy claims | % |
|  | Adherence to antidiabetics | Pharmacy claims | % |
